# Health-Related Quality of Life (HRQoL) changes in South Australia: comparison of burden of disease morbidity and survey-based health utility estimates

**DOI:** 10.1186/s12955-014-0113-4

**Published:** 2014-08-05

**Authors:** David Banham, Graeme Hawthorne, Robert Goldney, Julie Ratcliffe

**Affiliations:** Wardliparingga Aboriginal Health Research, South Australian Health and Medical Research Institute, North Terrace, Adelaide, SA 5000 Australia; Discipline of Public Health, School of Population Health, University of Adelaide, Terrace Towers, North Terrace, Adelaide, SA 5000 Australia; Mental Health Evaluation Unit, Department of Psychiatry, The University of Melbourne, Level 1 North, Royal Melbourne Hospital, Parkville, VIC, 3050 Australia; Emeritus Professor, Discipline of Psychiatry, University of Adelaide, Level 4, Eleanor Harrald Building Royal Adelaide Hospital, Adelaide, SA 5000 Australia; Flinders Clinical Effectiveness, School of Medicine, Flinders University, Room 52, A Block, Repatriation General Hospital, Daws Road, Daw Park, Adelaide, South Australia 5041 Australia

**Keywords:** Health related quality of life, Morbidity, Population health, Burden of disease, Health utility, Patient reported outcome measures, AQoL

## Abstract

**Background:**

Global research shows a clear transition in health outcomes over the past two decades where improved survival was accompanied by lower health related quality of life (HRQoL) as measured by morbidity and disability. These trends suggest the need to better understand changes in population HRQoL. This paper compares two perspectives on population HRQoL change using burden of disease morbidity estimates from administrative data and self-reports from random and representative population surveys.

**Methods:**

South Australian administrative data including inpatient hospital activity, cancer and communicable disease registrations were used within a Burden of Disease study framework to quantify morbidity as Prevalent Years of Life lived with Disease and injury related illness (PYLD) for 1999 to 2008. Self-reported HRQoL was measured using the Assessment of Quality of Life (AQoL) in face to face interviews with at least 3000 respondents in each of South Australia’s Health Omnibus Surveys (HOS) in 1998, 2004 and 2008.

**Results:**

Age specific PYLD rates for those aged 75 or more increased by 5.1%. HRQoL dis-utility in this age group also increased significantly and beyond the minimally important difference threshold. Underlying increased dis-utility were greater difficulties in independent living (particularly requiring help with household tasks) and psychological well-being (as influenced by pain, discomfort and difficulty sleeping).

**Conclusions:**

Consistent with increased quantity of life being accompanied by reduced HRQoL, the analysis indicates older people in South Australia experienced increased morbidity in the decade to 2008. The results warrant routine monitoring of health dis-utility at a population level and improvement to the supply and scope of administrative data.

## Background

A health system’s fundamental aim is to maintain or improve health in a given community. While changes in a population’s health are often expressed in terms of quantity, using life expectancy or mortality rates, such measures overlook health related quality of life (HRQoL). The latter is increasingly important to monitor as recent international research shows a clear transition in health outcomes over the past two decades whereby improved survival was accompanied by lower HRQoL as measured by morbidity and disability [1,2]. A similar analysis of South Australian data for the decade to 2008 also indicated health gains are being achieved overall [3], albeit increased life expectancy was accompanied by a relative expansion in disease and injury related morbidity. This corresponded with lower HRQoL in the South Australian population as a whole, particularly in older age groups [3] and as a result of increased prevalence of chronic conditions [4].

These trends suggest the need to better understand changes in population HRQoL. For a health system responding to changing health need while operating in a strict budget context it also means demonstrating how service activity and resource use best contribute to maintaining and increasing HRQoL during longer survival among patients and the wider population. Quantifying HRQoL requires summary measures that enable description and comparison of HRQoL within and across populations [5].

In quantifying non-fatal, morbid health outcomes for a wide range of diseases and injuries, the burden of disease framework combines epidemiological parameters and severity weights for different health states [6]. Relevant South Australian summary measures of population health (SA SMPH) [7] are available for the period 1999 to 2008. These are based on the general framework of the Australian Burden of Disease and Injury study [8] and an extensive range of local jurisdiction administrative unit records and survey estimates as detailed on the study website [7]. Despite the rigour of this work, the results are limited in capturing changes in underlying morbid disease. This is partly because routine data supply is lacking in some major condition areas such as mental health, which account for around 20% of total morbidity, and health sectors, such as primary care. Burden of disease studies also contain uncertainty from imprecision in parameters such as prevalence data and severity weighting [3,8,9]. None of the studies published before 2012 quantify the statistical uncertainty around estimates [9,10]. While it is now feasible to do so using simulation methods [8,10,11], this was also beyond the scope of the SA SMPH series because of the resourcing challenges associated with developing then maintaining condition-specific models [8], particularly for a single jurisdiction [3].

Nonetheless, burden of disease measures can be generally helpful in a policy and planning environment by objectively scoping population needs associated with particular conditions and risk factors [12]. It is also possible to use these to examine the macro-influence of interventions on society [3,13]. However, these measures do not directly link with operational health service outcomes, for example in evaluating the cost effectiveness and quality of program and treatment outcomes from a client perspective [13].

Self-reported health status measures provide a subjective, personalised assessment of the burden of disease and treatment experience without necessarily focussing on aetiology [14]. Health utility measures offer such a perspective on HRQoL, and in a manner specifically intended for evaluating healthcare treatment and service programmes via cost-utility analyses [15]. South Australia’s Health Omnibus Survey (HOS) is a random and representative household survey. The HOS first included a dedicated health utility measure in 1998 with repeats in 2004 and 2008.

This situation provides an opportunity to undertake novel comparison of two different perspectives on HRQoL based on administrative records of illness within burden of disease morbidity estimates and individual, self-reported health utility in the same population at very similar time points. With the aim of examining the relationship between the two approaches and the extent to which they harmonise, this paper:

- Describes repeated cross-sectional perspectives on HRQoL in South Australia’s adult population;
- Examines patterns of change across the decade to 2008; and
- Considers the health dimensions underlying observed changes.

The knowledge gained will inform discussions of appropriate methods for ongoing monitoring of population HRQoL while also informing and evaluating relevant service responses.

## Methods

### Burden of disease

The Burden of Disease and Injury in Australia, 2003 study [8] provides the base descriptive epidemiology and outcome estimates for SA SMPH. The South Australian series adjusts morbidity parameters according to yearly changes in sex and age groups for conditions observed in routinely available administrative data. Annually updated data include: unit records for cancer registrations, birth defects, communicable diseases, sexually transmitted infections; and, inpatient activity in South Australian hospitals.

#### Measuring amount and severity of prevalent disease and injury related illness

The burden of disease method represents morbidity as life lived in less than full health because of disease and injury related conditions. A given condition’s prevalent morbidity is expressed as Prevalent Years of Life lived with Disease and injury related illness (PYLD), the product of prevalence, severity weighting and duration (if duration is less than one year). Total population morbidity is PYLD summed across all conditions. Coupled with resident population estimates, the method provides morbidity rates for sex and age groups. A per person rate of 0 indicates no morbidity, with increasing rates meaning increasing morbidity; a value of 1.00 indicates total morbidity, or death.

#### Participants

The analysis uses PYLD estimates for ages 15 years and above for the years 1999, 2004 and 2008. The annually updated data underlying these estimates includes a yearly minimum of 540 000 inpatient hospitalisations and 7 200 cancer registrations. Use is also made of periodic South Australian prevalence data from cross-sectional population surveys of approximately 7 000 people each year [16].

### Population-based self-reported HRQoL

South Australia’s HOS is an annual, cross-sectional, face-to-face, random and representative survey. Each survey samples households using a clustered, multi-staged and self-weighted area design yielding at least 3 000 interviews of persons aged 15 years or more, living in the Adelaide metropolitan area or townships of at least 1 000 people [17].

#### Measuring health dis-utility

The Assessment of Quality of Life (AQoL) instrument [18] comprises 15 items. Each item response is coded 0 to 3 (indicating best through to worst status) and can be considered separately or summed to form a simple, unweighted profile [19]. The items cover five HRQoL dimensions of: illness; independent living; social relationships; physical senses; and psychological wellbeing. Each dimension yields a weighted dis-utility score from 0 (worst) to 1 (best) which can be assessed for change *within* each dimension but not *across* dimensions [19]. Using Australian general population values based on the time trade-off elicitation technique, the latter four dimensions combine multiplicatively for an overall utility score ranging from worst possible (-0.04) (e.g. where a person wishes to die immediately), death equivalent (0.00) to full HRQoL (1.00) [19]. Change of 0.06 (95% CI: 0.03 – 0.08) or more represents a ‘minimally important difference’ (MID) in patient populations [20] warranting change to an individual’s health treatment [21] in a clinical setting. In establishing this MID threshold, 20% of respondents were recovering from emergency (life-threatening, high dis-utility) situations and reported higher thresholds (around 0.13). The majority had chronic conditions in community settings and reported lower thresholds in the order of 0.03. Nevertheless, the 0.06 MID threshold was adopted as a conservative estimate for the current analysis focussed on a population-wide setting.

To facilitate comparison between PYLD and utility scores, the latter was reflected into dis-utility scores (where dis-utility = 1- utility score) and rescaled so all scores were in the range 0 to 1 [19]. This means 0 consistently represents full HRQoL, an increasing dis-utility score indicates decreasing HRQoL and increasing morbidity, and 1 represents worst possible HRQoL.

#### Participants

Responses for a total of 9059 participants were available from the 1998, 2004 and 2008 HOS (N = 3010, 3015 and 3034 respectively).

SA Health’s Human Research Ethics Committee approved the secondary analysis of these data (314/08/2012).

### Data analysis

Crude population morbidity rates are initially reported for each time period. Over time the underlying structure of the population changed and from 1998 to 2008 South Australia’s population aged 75 years or more increased by 27,800 from 6.3% to 7.6% of total population. To account for these changes sex and age rates were directly adjusted to the Australian population as at 30 June 2001 as recommended by the Australian Bureau of Statistics [22] and calculated for each time period. This was achieved by applying the observed sex and age specific PYLD rates to the Australian standard population, then dividing the sum by the total standard population.

HOS data files include weights matching the Australian Bureau of Statistics Estimated Residential Population data for South Australia in each individual year and are designed for reporting results for 5 year age groups by sex and crude, total population outcomes. Sex and age standardised comparisons across surveys were enabled using a purpose designed re-weighting algorithm (G. Tucker, Health Statistics Unit, Epidemiology, SA Health, pers. comm., 5 July 2011).

Arithmetic means are reported for easier interpretation and discussion of descriptive results. Hence, positively skewed dis-utility and dimension outcomes are not reflected and log transformed before regression analysis [23]. While the F test is robust to departures from normality [24], a conservative approach to analysis is nonetheless adopted and robust standard errors computed to minimise assumptions about the data and subsequent linear regression models [25]. All analyses were conducted with Stata version 12 [26]. To guide the analyses toward population outcomes warranting further discussion and response, several ex ante criteria were adopted, viz:

- 1. Mean change across the three available periods must be incremental and in the same direction. For example, if it is true that PYLD and/or AQoL dis-utility were higher in 2008 than 2004 and 2004 was higher than 1998, then HRQoL is assessed as having decreased over time;
- 2. Mean change assessed must be statistically significant. That is, there was a significant difference between the highest and lowest HRQoL outcomes observed across the three time periods;
- 3. Mean change must exceed the published MID threshold.

## Results

The population samples interviewed are detailed separately [27] but, in short, HOS 1998 yielded 3010 interviews with 82% of those in scope participating, 3015 (76% participation) in 2004 and 3014 interviews (73% participation) in 2008. AQoL dis-utility scores were available for 99.2% (8974) of responses.

Mean results for PYLD and the AQoL across the three time periods and assessment against the three guiding criteria are summarised in Table 1.

**Table 1:** **HRQoL by Year and population stratum**

**Year**																			
	**1998/1999**				**2004**				**2008**				Incremental change*		**Significant change across years**		**Minimally important**	**Absolute change**	**Relative change**
**Measure**	**Percent population**	**Mean**	***L95% CI***	***U95% CI***	**Percent population**	**Mean**	***L95%CI***	***U95%CI***	**Percent population**	**Mean**	***L95% CI***	***U95% CI***	***2004 > 1998***	***2008 > 2004***		***p***	**change**		**(%)**
PYLD rate per person																			
Crude	100%	0.105	n.a.	n.a.	100%	0.108	n.a.	n.a.	100.0%	0.110	n.a.	n.a.	TRUE	TRUE	n.a.	n.a.	n.a.	0.005	5.0%
Standardised																			
Persons		0.102	*n.a.*	*n.a.*		0.102	*n.a.*	*n.a.*		0.102	*n.a.*	*n.a.*	TRUE	TRUE	n.a.	n.a.	n.a.	0.000	0.3%
Male		0.102	*n.a.*	*n.a.*		0.103	*n.a.*	*n.a.*		0.103	*n.a.*	*n.a.*	TRUE	TRUE	n.a.	n.a.	n.a.	0.001	0.9%
Female		0.101	*n.a.*	*n.a.*		0.101	*n.a.*	*n.a.*		0.101	*n.a.*	*n.a.*	TRUE	FALSE	n.a.	n.a.	n.a.	0.000	-0.2%
Age																			
15-24	16.8%	0.043	*n.a.*	*n.a.*	16.5%	0.042	*n.a.*	*n.a.*	16.6%	0.042	*n.a.*	*n.a.*	FALSE	TRUE	n.a.	n.a.	n.a.	0.000	-0.7%
25-34	17.8%	0.063	*n.a.*	*n.a.*	16.1%	0.064	*n.a.*	*n.a.*	15.4%	0.062	*n.a.*	*n.a.*	TRUE	FALSE	n.a.	n.a.	n.a.	-0.002	-2.6%
35-44	19.0%	0.072	*n.a.*	*n.a.*	18.1%	0.073	*n.a.*	*n.a.*	17.2%	0.070	*n.a.*	*n.a.*	TRUE	FALSE	n.a.	n.a.	n.a.	-0.002	-3.0%
45-54	17.0%	0.092	*n.a.*	*n.a.*	17.4%	0.093	*n.a.*	*n.a.*	17.3%	0.092	*n.a.*	*n.a.*	TRUE	FALSE	n.a.	n.a.	n.a.	-0.001	-0.7%
55-64	11.5%	0.125	*n.a.*	*n.a.*	13.6%	0.124	*n.a.*	*n.a.*	14.7%	0.126	*n.a.*	*n.a.*	FALSE	TRUE	n.a.	n.a.	n.a.	0.001	1.1%
65-74	9.7%	0.183	*n.a.*	*n.a.*	9.2%	0.183	*n.a.*	*n.a.*	9.4%	0.184	*n.a.*	*n.a.*	FALSE	TRUE	n.a.	n.a.	n.a.	0.000	0.1%
75+	8.2%	0.304	*n.a.*	*n.a.*	9.1%	0.308	*n.a.*	*n.a.*	9.3%	0.320	*n.a.*	*n.a.*	TRUE	TRUE	n.a.	n.a.	n.a.	0.015	5.1%
																	Threshold		
AQoL dis-utility**																	=0.060		
Crude		0.167	*0.160*	*0.175*		0.183	*0.176*	*0.191*		0.199	*0.191*	*0.207*	TRUE	TRUE	F(1, 8972)=29.31	<0.001	FALSE	0.032	18.8%
Standardised																			
Persons		0.183	0.175	0.190		0.198	0.191	0.214		0.214	0.214	0.231	TRUE	TRUE	F(1, 8972)=30.60	<0.001	FALSE	0.031	17.2%
Male		0.179	0.167	0.190		0.183	0.173	0.194		0.212	0.200	0.224	TRUE	TRUE	F(1, 3756)=13.71	<0.001	FALSE	0.033	18.8%
Female		0.186	0.176	0.196		0.213	0.203	0.223		0.216	0.206	0.226	TRUE	TRUE	F(1, 5214)=18.66	<0.001	FALSE	0.030	16.0%
Age																			
15-24		0.128	0.109	0.147		0.126	0.108	0.144		0.129	0.112	0.146	FALSE	TRUE	F(1, 1022)=0.00	0.99	FALSE	0.001	0.4%
25-34		0.135	0.116	0.154		0.151	0.134	0.169		0.143	0.125	0.161	TRUE	FALSE	F(1, 1362)=0.06	0.45	FALSE	0.008	5.9%
35-44		0.149	0.132	0.166		0.148	0.134	0.162		0.180	0.162	0.198	FALSE	TRUE	F(1, 1717)=5.14	0.02	FALSE	0.031	20.8%
45-54		0.161	0.143	0.179		0.193	0.175	0.211		0.215	0.194	0.235	TRUE	TRUE	F(1, 1451)=15.09	<0.001	FALSE	0.054	33.3%
55-64		0.204	0.183	0.225		0.223	0.201	0.246		0.236	0.214	0.258	TRUE	TRUE	F(1, 1366)=4.37	0.04	FALSE	0.032	15.9%
65-74		0.211	0.189	0.232		0.220	0.198	0.243		0.222	0.200	0.245	TRUE	TRUE	F(1, 1074)=0.54	0.47	FALSE	0.011	5.3%
75+		0.291	0.257	0.325		0.300	0.274	0.327		0.351	0.319	0.383	TRUE	TRUE	F(1, 968)=5.84	0.02	TRUE	0.060	20.7%

*Where it is true that PYLD rates and/or AQoL dis-utility were higher in 2008 than 2004, and 2004 was higher than 1998, then HRQoL is considered to have reduced.

**Rescaled so all dis-utility scores are in the range 0 to 1.

PYLD based assessment of crude morbidity for the population aged 15 or more, increased from years 1999 to 2004 and from 2004 to 2008 for a relative rate increase of 5.0% overall. After standardising for sex and age changes over time, the movement was negligible at 0.3%. Similarly, there were small changes in age standardised outcomes for males and females overall. However, the 75 years or more age category was associated with increases in PYLD rates from baseline to 2004 and again to 2008, a relative increase of 5.1% across the decade. The statistical significance of this cannot be formally assessed [8], nor is there an established, important threshold of change to compare it with.

Total HRQoL dis-utility in the population measured by the AQoL increased across consecutive time periods from 1998 to 2004 and again to 2008 with an 18.8% (16.7% to 19.9%) increase. While statistically significant, this did not reach the MID threshold. Similarly, standardised dis-utility outcomes for total population, males and females separately each increased incrementally and at a statistically significant level over time. However, none of these changes reached the MID.

The 75 and over age group had dis-utility results which met all three criteria. That is: dis-utility increased from 1998 to 2004 and 2004 to 2008; the change from baseline to 2008 was statistically significant and the increase reached the MID. Those aged 45-54 reported significantly higher dis-utility between 1998 and 2004, but not between 2004 and 2008. The overall (1998-2008) increase for these participants met the MID threshold (an absolute change of 0.06) [20]. While not reaching the MID threshold, respondents aged 55 to 74 reported incrementally increased dis-utility over time with statistically significant increases among those aged 55-64 years.

Table 2 summarises the health dimensions assessed by AQoL and their underlying items for respondents aged 75 and over. Illness, as assessed by higher consumption of medical aids, increased across consecutive time periods and significantly so from 1998 to 2008. Two items within the Illness dimension had similar patterns of change, with prescribed medicine use and need for medical treatment both increasing significantly. Illness does not directly load into dis-utility scores but two dimensions that do had significant, incremental increases amounting to one-third extra morbidity. The Independent Living dimension was particularly influenced by respondents indicating the need for more help in doing household tasks. Increased pain or discomfort and to a lesser extent, interruptions to sleep, contributed to lower Psychological Wellbeing.

**Table 2:** **AQoL dimension and item results in ages 75 or more**

**Dimension dis-utility (0 = best; 1 = worst)**	**Item (0 = best to 3 = worst)**	**1998**			**Year 2004**			**2008**			Incremental change*		**Significant change across 3 time periods**		**Absolute change**	**Relative change (%)**
		**Mean**	**L95%CI**	**U95%CI**	**Mean**	**L95%CI**	**U95%CI**	**Mean**	**L95%CI**	**U95%CI**	**2004 > 1998**	**2008 > 2004**		***p***	**(from 1998 to 2008)**	
Illness		0.496	0.455	0.538	0.637	0.605	0.668	0.644	0.609	0.678	TRUE	TRUE	F(1, 967) = 30.35	0.000	0.147	29.6%
	1. Use of prescribed medicines	1.257	1.129	1.385	1.675	1.566	1.783	1.822	1.708	1.937	TRUE	TRUE	F(1, 968) = 42.88	0.000	0.565	25.1%
	2. Reliance on medicines or medical aids	1.589	1.434	1.744	2.206	2.103	2.309	2.186	2.072	2.299	TRUE	FALSE	F(1, 960) = 39.02	0.000	0.596	23.0%
	3. Need for medical treatment	1.138	1.000	1.276	1.316	1.196	1.437	1.384	1.256	1.513	TRUE	TRUE	F(1, 968) = 6.80	0.009	0.246	11.5%
Independent Living		0.145	0.115	0.174	0.157	0.131	0.183	0.189	0.157	0.220	TRUE	TRUE	F(1, 968) = 3.78	0.052	0.044	30.2%
	4. Help with personal care	0.245	0.161	0.329	0.214	0.143	0.286	0.333	0.246	0.420	FALSE	TRUE	F(1, 968) = 1.70	0.192	0.088	7.0%
	5. Help with household tasks	0.674	0.552	0.797	0.749	0.642	0.856	0.922	0.796	1.049	TRUE	TRUE	F(1, 969) = 7.33	0.007	0.248	14.8%
	6. Getting around home and community	0.341	0.248	0.434	0.390	0.301	0.480	0.456	0.348	0.565	TRUE	TRUE	F(1, 968) = 2.51	0.114	0.115	8.6%
Social Relations		0.083	0.062	0.104	0.077	0.062	0.093	0.101	0.082	0.121	FALSE	TRUE	F(1, 968) = 1.28	0.258	0.018	22.0%
	7. Warmth of personal relationships	0.213	0.141	0.128	0.210	0.159	0.262	0.293	0.220	0.366	FALSE	TRUE	F(1, 966) = 2.13	0.145	0.080	37.7%
	8. Relationships with others	0.349	0.272	0.427	0.312	0.251	0.373	0.406	0.329	0.484	FALSE	TRUE	F(1, 968) = 0.83	0.364	0.057	16.4%
	9. Relationship with family	0.216	0.147	0.285	0.246	0.181	0.311	0.293	0.224	0.363	TRUE	TRUE	F(1, 964) = 2.33	0.128	0.077	35.7%
Physical Senses		0.079	0.065	0.094	0.074	0.062	0.086	0.084	0.070	0.097	FALSE	TRUE	F(1, 968) = 0.11	0.738	0.004	5.2%
	10. Vision	0.366	0.285	0.446	0.304	0.233	0.374	0.349	0.268	0.431	FALSE	TRUE	F(1, 968) = 0.13	0.714	-0.016	-4.4%
	11. Hearing	0.437	0.355	0.520	0.457	0.383	0.530	0.500	0.419	0.582	TRUE	TRUE	F(1, 967) = 1.09	0.297	0.063	14.4%
	12. Communication	0.174	0.110	0.239	0.127	0.081	0.173	0.158	0.102	0.214	FALSE	TRUE	F(1, 968) = 0.22	0.640	-0.017	-9.6%
Psychological Wellbeing		0.099	0.084	0.114	0.101	0.089	0.112	0.132	0.114	0.150	TRUE	TRUE	F(1, 968) = 6.88	0.009	0.033	33.0%
	13. Sleep patterns	0.805	0.680	0.930	0.917	0.808	1.026	0.966	0.840	1.092	TRUE	TRUE	F(1, 968) = 3.25	0.072	0.161	8.9%
	14. Affective feelings	0.368	0.286	0.450	0.259	0.199	0.319	0.427	0.349	0.505	FALSE	TRUE	F(1, 969) = 0.61	0.434	0.059	4.3%
	15. Pain or discomfort	0.600	0.521	0.678	0.652	0.582	0.722	0.773	0.691	0.855	TRUE	TRUE	F(1, 968) = 8.62	0.003	0.173	10.8%

*Where the AQoL dis-utility dimension was higher in 2008 than 2004, and 2004 was higher than 1998, then HRQoL is considered to have reduced.

Results for the 75 and over age group are based on 970 responses (282 for ages 75 to 79 years; 354 for 80 to 84; 334 for 85 or more). While not supporting detailed analysis of these narrower age groups across time, a broad overview is warranted (Table 3). Respondents aged 75 to 79 had the largest age increase of 0.4 years to 77.0 years in 2008 but the smallest dis-utility increase, 0.006. Conversely, those aged 80 to 84 had the smallest age increase with 0.1 year to 81.8 by 2008 while reporting the largest dis-utility increase of 0.107.

**Table 3:** **Age and AQoL dis-utility changes within ages 75 or more**

	**1998**			**2004**			**2008**			**Change**		
										**(1998 to 2008)**		
	Mean	L95% CI	U95% CI	Mean	L95% CI	U95% CI	Mean	L95% CI	U95% CI	Mean	L95% CI	U95% CI
Age												
75-79	76.7	76.4	76.9	77.0	76.8	77.2	77.0	76.8	77.3	0.4	0.3	0.4
80-84	81.7	81.3	82.1	81.7	81.5	82.0	81.8	81.5	82.1	0.1	0.0	0.2
85+	87.8	86.9	88.7	87.5	86.9	88.2	88.0	87.2	88.7	0.1	0.0	0.3
AQoL dis-utitlity												
75-79	0.265	0.222	0.307	0.276	0.241	0.311	0.270	0.233	0.308	0.006	0.001	0.011
80-84	0.286	0.225	0.346	0.289	0.242	0.336	0.393	0.337	0.449	0.107	0.103	0.111
85+	0.408	0.307	0.508	0.423	0.346	0.501	0.484	0.403	0.565	0.076	0.057	0.096

## Discussion

Burden of disease and health dis-utility perspectives each indicate lower HRQoL among adult South Australians in the decade to 2008. This is not unexpected given the community’s age profile as a whole is changing and South Australia has a higher proportion of people in older age categories than most other Australian states and territories [28]. Nevertheless, when these sex and age changes were allowed for, increased PYLD and dis-utility rates persisted, albeit in smaller amounts in PYLD. Where formal testing is possible, these increases were statistically significant but not sufficiently large to exceed the MID and justify a whole of population response.

Changes at the whole of (adult) population level were influenced by variations in several age groups. Consistent with international studies [2], the largest absolute change observed in age specific HRQoL was among those aged 75 or more. In this particular age group PYLD and dis-utility measures both describe increased morbidity and lower HRQoL. Formal appraisals of changed dis-utility were not only statistically significant among those aged 75 or more, but they were also above the ‘minimally important’ threshold for change. Given the population of older adults has higher rates of people with health conditions, this may in part explain the study findings. Indeed, the AQoL threshold was calculated on small patient samples who were usually older and represented a narrow range of chronic, health conditions. While no published evidence directly generalise from ‘minimally important’ thresholds in patient samples to population-based cohorts, this study’s findings assessed change using conservative criteria and suggest the need for monitoring HRQoL change in older age to inform and monitor relevant service responses.

The AQoL enables scrutiny of change within several health dimensions. Survey respondents aged 75 or more years reported increased trouble in maintaining levels of independent living, particularly in requiring more help with household tasks; and psychological well-being as influenced by experiences of pain, discomfort and difficulty sleeping. Respondents also reported greater use of prescribed medications and increased dependence on medical treatment from health professionals.

Reports of increased morbidity in older ages across time are consistent with analysis of healthy life expectancy change in South Australia which shows that considerable improvement in mortality rates among older people has not fully translated into healthy life expectancy gains [3]. By inference, these findings indicate a relative expansion of morbidity during the decade to 2008. The current analysis uses the same (SA SMPH) data to describe a small, absolute change in morbidity, as indicated by higher PYLD rates in older age. While this is based on administrative records, the validity of this increased morbidity is reinforced by subjective, self-reported assessment among older people living in the community.

In other age groupings, changes in dis-utility experience differed markedly from the PYLD perspective. For example, ages 55-64 reported significantly increased morbidity albeit this did not reach the MID threshold. Nevertheless, changes in dis-utility reports were uniformly larger than those described by PYLD.

One known contributor to a relative insensitivity to change in PYLD is that annual estimates are not adequately informed on important morbidity issues. Yet this is an important area as mental health conditions account for one in every five years lost to prevalent illness and successive waves of a South Australian cohort confirm the complex interplay between mental health issues, chronic physical conditions and lifestyle risks, particularly among the middle aged [4]. The lack of routinely available data in this area is a notable limitation in monitoring morbidity change over time. The extent of changed psychological-wellbeing reported by older survey respondents is consistent with this. Interestingly, change on this health dimension was influenced by sleep patterns and pain rather than affective feelings per se. This is important, as it may suggest that psychological health deterioration in older adults is a function of deteriorating physical health as much as an independent psychological phenomenon. Nevertheless, the important role of psychological wellbeing in respondents’ self-reporting of health resonates with other recent Australian literature in which older people with multiple chronic conditions report emotional well-being is a pressing issue, but one not always addressed [29].

Another plausible explanation for differences in PYLD and dis-utility outcomes is that middle aged, or baby boomer, survey respondents reported the effects of early stage, sub-clinical conditions, such as pre-diabetes and/or increased risk factor exposures, for example elevated body mass and sedentary behaviours [4]. Where conditions are yet to manifest, or are managed in primary care settings, they are not likely to be included in the available administrative records.

One final explanation also requires monitoring and exploration into the future. If health care budgets are constrained, yet the health of a given population deteriorates, then over time there will be an increasing disconnect between the PYLD burden of disease estimate and self-reported HRQoL. If this is true, then there are important sequelae, particularly where burden of disease estimates are derived from records of health service use rather than population surveys.

Gender differences in PYLD and dis-utility results are also apparent with PYLD rates higher among males while dis-utility levels on the AQoL are higher among females. The burden of disease method uses discrete gender and age specific severity weights for each condition and its sequelae [8]. Thus, changes in PYLD rate reflect variation in the amount of prevalent disease and injury within those conditions and their sequelae by sex and age. Any inter-relationships between a patient, their clinical condition and context are not accounted for [30]. On the other hand, dis-utility instruments enable respondents to report their subjective, functional experience from within their particular life context. This can include disease and injury related morbidity while also accounting for the influences of bearing care-giving roles for example. Raised awareness and new knowledge may also influence subjective self-reporting. For example, increased mental health literacy has been accompanied by increased reports of depression which may suggest extra knowledge promotes introspection and endorsement of symptoms [27]. One further potential issue for subjective, self-reported HRQoL is adaptation or adjustment to changed states [31], particularly among older people. For example, observation of clinical populations show that a proportion of people who clearly have significant mobility impairments refuse to report problems in walking about and rate themselves at perfect health [32]. Together with the age and self-reported dis-utility changes within this study’s older age group this suggests further detailed examination of HRQoL within older ages is required.

The use of three sets of cross-sectional data across a decade could be regarded as a limitation. However the underlying data and analysis have compensating strengths, one of which is the sourcing of information acquired through well developed and rigorous methods. For example, the HOS face-to-face interviews yield a highly regarded and widely used population-representative data source [17]. Replicate cross-sectional surveys also take into account changes over time in the underlying structure of the population of interest. Also, Australia’s approach to conducting burden of disease studies has been the subject of scrutiny and review over a lengthy time [8] and the burden framework continues to evolve. For example, technical infrastructure developments in the most recent Global Burden of Disease update [1,6] now make routine calculation of uncertainty estimates for PYLD increasingly feasible within national projects [11]. Consequently, it is anticipated that Australia’s forthcoming updates will include PYLD uncertainty intervals and account for measurable error such as the relative standard errors within survey data and error from meta-analyses [10]. These improvements will overcome one of the limitations experienced by this current analysis.

## Conclusions

Comparison of two different perspectives on HRQoL both point to increased morbidity among older persons, but do not explain why that increase took place. Dis-utility measures also suggest a trend toward increasing morbidity in older middle-age but this is not reflected in PYLD results, the latter appearing less sensitive to change generally.

The results warrant routine monitoring of health dis-utility at a population level in concert with improved supply and scope of administrative data. Merging administrative records and self-reported measures into linked, person-centred datasets as occurs in clinical studies, would enhance description of outcomes for patient groups and populations [33,34]. In turn, this will better inform discussion about improving population morbidity, the influence of health service activities on HRQoL outcomes and ultimately, improved healthy life expectancy.

## References

[1] Salomon JA, Wang H, Freeman MK, Vos T, Flaxman AD, Lopez AD, Murray CJL (2012). Healthy life expectancy for 187 countries, 1990–2010: a systematic analysis for the Global Burden Disease Study 2010. Lancet.

[2] Audureau E, Rican S, Coste J (2013). Worsening trends and increasing disparities in health-related quality of life: evidence from two French population-based cross-sectional surveys, 1995-2003. Qual Life Res.

[3] Banham D, Woollacott T, Lynch J (2011). Healthy life gains in South Australia 1999-2008: analysis of a local Burden of Disease series. Popul Health Metrics.

[4] Buckley J, Tucker G, Hugo G, Wittert G, Adams RJ, Wilson DH (2013). The Australian Baby Boomer Population—Factors Influencing Changes to Health-Related Quality of Life Over Time. J Aging Health.

[5] Brock DW, Anand S, Peter F, Sen A (2004). Ethical issues in the use of cost effectiveness analysis for the prioritisation of health care resources. Public Health, Ethics, and Equity.

[6] Vos T, Flaxman AD, Naghavi M, Lozano R, Michaud C, Ezzati M, Shibuya K, Salomon JA, Abdalla S, Aboyans V, Abraham J, Ackerman I, Aggarwal R, Ahn SY, Ali MK, Alvarado M, Anderson HR, Anderson LM, Andrews KG, Atkinson C, Baddour LM, Bahalim AN, Barker-Collo S, Barrero LH, Bartels DH, Basáñez M-G, Baxter A, Bell ML, Benjamin EJ, Bennett D (2012). Years lived with disability (YLDs) for 1160 sequelae of 289 diseases and injuries 1990–2010: a systematic analysis for the Global Burden of Disease Study 2010. Lancet.

[7] ᅟ: **South Australian Burden of Disease website.** In *ᅟ.* ; ᅟ. [www.sahealth.sa.gov.au/burdenofdisease] Accessed 19 June 2014.

[8] Begg S, Vos T, Barker B, Stevenson C, Stanley L, Lopez AD (2007). Burden of Disease and Injury in Australia, 2003.

[9] Polinder S, Haagsma J, Stein C, Havelaar A (2012). Systematic review of general burden of disease studies using disability-adjusted life years. Popul Health Metrics.

[10] Australian Institute of Health and Welfare (2014). Assessment of Global Burden of Disease 2010 methods for the Australian context: Australian Burden of Disease Study Working paper No. 1.

[11] Ministry of Health (New Zealand) (2012). Ways and Means: A report on methodology from the New Zealand Burden of Diseases, Injuries and Risk Factors Study, 2006–2016.

[12] Struijk EA, May AM, Beulens JWJ, de Wit GA, Boer JMA, Onland-Moret NC, van der Schouw YT, Bueno-de-Mesquita HB, Hoekstra J, Peeters PHM (2013). Development of Methodology for Disability-Adjusted Life Years (DALYs) Calculation Based on Real-Life Data. PLoS One.

[13] Banham D, Lynch J, Karnon J (2011). An equity–effectiveness framework linking health programs and healthy life expectancy. Aust J Prim Health.

[14] Calvert M, Brundage M, Jacobsen P, Schunemann H, Efficace F (2013). The CONSORT Patient-Reported Outcome (PRO) extension: implications for clinical trials and practice. Health Qual Life Outcomes.

[15] Brazier J, Ratcliffe J, Salomon JA, Tsuchiya A (2007). Measuring and valuing health benefits for economic evaluation.

[16] Population Research and Outcome Studies (2004). South Australian Monitoring and Surveillance System (SAMSS): Survey methodology. SAMSS Technical Paper Series.

[17] Taylor A, Dal Grande E, Wilson D (2006). The South Australian Health Omnibus Survey 15 Years on: Has Public Health Benefited?. Public Health Bull (S Aust).

[18] Hawthorne G, Richardson J, Osborne R (1999). The Assessment of Quality of Life (AQoL) instrument: a psychometric measure of health-related quality of life. Qual Life Res.

[19] Hawthorne G, Richardson J, Day N (2000). Using the Assessment of Quality of Life (AQoL) Instrument - Version 1.0. The Centre for Health Program Evaluation.

[20] Hawthorne G, Osborne R (2005). Population norms and meaningful differences for the Assessment of Quality of Life (AQoL) measure. Aust N Z J Public Health.

[21] Walters SJ, Brazier JE (2005). Comparison of the minimally important difference for two health state utility measures: EQ-5D and SF-6D. Qual Life Res.

[22] Australian Bureau of Statistics (2013). Australian Demographic Statistics.

[23] Tabachnick BG, Fedele LS (1996). Using Multivariate Statistics.

[24] Kutner MH, Nachtsheim CJ, Neter J, Li W (2005). Applied Linear Statistical Models.

[25] Angrist JD, Pischke J-S (2008). Mostly harmless econometrics: An Empiricist’s Companion.

[26] StataCorp (2012). Stata Statistical Software: Release 12.

[27] Goldney RD, Dunn KI, Dal Grande E, Crabb S, Taylor A (2009). Tracking depression-related mental health literacy across South Australia: a decade of change. Aust N Z J Psychiatry.

[28] Australian Bureau of Statistics (2012). Yearbook Australia 2012.

[29] Gilbert AL, Caughey GE, Vitry AI, Clark A, Ryan P, McDermott RA, Shakib S, Luszcz MA, Esterman A, Roughead EE (2011). Ageing well: Improving the management of patients with multiple chronic health problems. Australas J Ageing.

[30] Reidpath DD, Allotey PA, Kouame A, Cummins RA (2003). Measuring health in a vacuum: examining the disability weight of the DALY. Health Policy Plan.

[31] King MT (2011). A point of minimal important difference (MID): a critique of terminology and methods. Expert Rev Pharmacoecon Outcomes Res.

[32] Ratcliffe J, Laver K, Couzner L, Crotty M, Atwood PC (2012). Health economics and geriatrics: challenges and opportunities. Geriatrics.

[33] McGrail K, Bryan S, Davis J (2012). Let’s All Go to the PROM: The Case for Routine Patient-Reported Outcome Measurement in Canadian Healthcare. Healthc Pap.

[34] Gutacker N, Bojke C, Daidone S, Devlin N, Street A (2013). Hospital Variation in Patient-Reported Outcomes at the Level of EQ-5D Dimensions: Evidence from England. Med Decis Making.

